# Evidence for Dual Activation of *I*_K(M)_ and *I*_K(Ca)_ Caused by QO-58 (5-(2,6-Dichloro-5-fluoropyridin-3-yl)-3-phenyl-2-(trifluoromethyl)-1H-pyrazolol[1,5-a]pyrimidin-7-one)

**DOI:** 10.3390/ijms23137042

**Published:** 2022-06-24

**Authors:** Chao-Liang Wu, Poyuan Fu, Hsin-Yen Cho, Tzu-Hsien Chuang, Sheng-Nan Wu

**Affiliations:** 1 Department of Medical Research, Ditmanson Medical Foundation Chia-Yi Christian Hospital, Chiayi 60002, Taiwan; wumolbio@mail.ncku.edu.tw; 2 Department of Ophthalmology, Ditmanson Medical Foundation Chia-Yi Christian Hospital, Chiayi 60002, Taiwan; 07141@cych.org.tw; 3 Department of Physiology, National Cheng Kung University Medical College, Tainan 70101, Taiwan; s36094083@gs.ncku.edu.tw (H.-Y.C.); s36091051@gs.ncku.edu.tw (T.-H.C.); 4 Institute of Basic Medical Sciences, National Cheng Kung University Medical College, Tainan 70101, Taiwan

**Keywords:** QO-58 (5-(2,6-dichloro-5-fluoropyridin-3-yl)-3-phenyl-2-(trifluoromethyl)-1H-pyrazolol[1,5-a]pyrimidin-7-one, 5-(2,6-dichloro-5-fluoro-3-pyridinyl)-3-phenyl-2-(trifluoromethyl)-pyrazolo[1,5-a]pyrimidin-7(4*H*)-one), M-type K^+^ current, voltage-dependent hysteresis, M-type K^+^ channel, Ca^2+^-activated K^+^ current, large-conductance Ca^2+^-activated K^+^ channel

## Abstract

QO-58 (5-(2,6-dichloro-5-fluoropyridin-3-yl)-3-phenyl-2-(trifluoromethyl)-1H-pyrazolol[1,5-a]pyrimidin-7-one) has been regarded to be an activator of K_V_7 channels with analgesic properties. However, whether and how the presence of this compound can result in any modifications of other types of membrane ion channels in native cells are not thoroughly investigated. In this study, we investigated its perturbations on M-type K^+^ current (*I*_K(M)_), Ca^2+^-activated K^+^ current (*I*_K(Ca)_), large-conductance Ca^2+^-activated K^+^ (BK_Ca_) channels, and *erg*-mediated K^+^ current (*I*_K(erg)_) identified from pituitary tumor (GH_3_) cells. Addition of QO-58 can increase the amplitude of *I*_K(M)_ and *I*_K(Ca)_ in a concentration-dependent fashion, with effective EC_50_ of 3.1 and 4.2 μM, respectively. This compound could shift the activation curve of *I*_K(M)_ toward a leftward direction with being void of changes in the gating charge. The strength in voltage-dependent hysteresis (V_hys_) of *I*_K(M)_ evoked by upright triangular ramp pulse (V_ramp_) was enhanced by adding QO-58. The probabilities of M-type K^+^ (K_M_) channels that will be open increased upon the exposure to QO-58, although no modification in single-channel conductance was seen. Furthermore, GH_3_-cell exposure to QO-58 effectively increased the amplitude of *I*_K(Ca)_ as well as enhanced the activity of BK_Ca_ channels. Under inside-out configuration, QO-58, applied at the cytosolic leaflet of the channel, activated BK_Ca_-channel activity, and its increase could be attenuated by further addition of verruculogen, but not by linopirdine (10 μM). The application of QO-58 could lead to a leftward shift in the activation curve of BK_Ca_ channels with neither change in the gating charge nor in single-channel conductance. Moreover, cell exposure of QO-58 (10 μM) resulted in a minor suppression of *I*_K(erg)_ amplitude in response to membrane hyperpolarization. The docking results also revealed that there are possible interactions of the QO-58 molecule with the KCNQ or K_Ca_1.1 channel. Overall, dual activation of *I*_K(M)_ and *I*_K(Ca)_ caused by the presence of QO-58 eventually may have high impacts on the functional activity (e.g., anti-nociceptive effect) residing in electrically excitable cells. Care must be exercised when interpreting data generated with QO-58 as it is not entirely KCNQ/K_V_7 selective.

## 1. Introduction

QO-58 (5-(2,6-dichloro-5-fluoropyridin-3-yl)-3-phenyl-2-(trifluoromethyl)-1*H*-pyrazolol[1,5-a]pyrimidin-7-one) has been demonstrated previously to be an opener of KCNQx (K_V_7x) channel [1,2,3,4]. It has been reported that this compound could increase the pain threshold of neuropathic pain in a rat model (i.e., chronic constriction injury of the sciatic nerve) [2]. It could also exercise anti-nociceptive action on inflammatory pain in rodents [5,6]. The ameliorating effects of this compound have been viewed to be closely linked to its activation of KCNQ (K_V_7) channels [4,7,8]. However, QO-40 (5-(chloromethyl)-3-(nathphalen-1-yl)-2-(trifluoromethyl)pyrazolo[1,5-a]pyrimidin-7(4*H*)-one), a compound structurally similar to QO-58, has been noticeably reported to stimulate the activity of large-conductance Ca^2+^-activated K^+^ (BK_Ca_) channels [9]. Therefore, whether and to what extent QO-58 is able to modify the amplitude or gating kinetics on different types of membrane ion currents remain to be not thoroughly explored.

It has been demonstrated that the KCNQ2, KCNQ3, or KCNQ5 encodes the core subunit of K_V_7.2, K_V_7.3, or K_V_7.5 channel. The enhanced activity of this family of voltage-gated K^+^ channels (KCNQx, K_V_7x, or K_M_ [M-type K^+^] channels) can generate macroscopic M-type K^+^ current (*I*_K(M)_), which is biophysically characterized by current activation upon low-threshold voltage [10,11]. Once being evoked during membrane depolarization, this type of K^+^ currents, which is susceptible to block by linopirdine, has been disclosed to exhibit a slowly activating and deactivating property as well to affect the bursting patterns in different types of neurons, and endocrine or neuroendocrine cells [12,13,14,15,16,17,18,19,20]. Moreover, targeting *I*_K(M)_ has been growingly thought to be an adjunctive regimen for the management of varying neurological, smooth muscle, or endocrine disorders which are closely linked to membrane hyperexcitability. These disorders include neuropathic pain and epilepsy [4,11,19,21,22,23,24,25,26,27,28,29,30,31,32,33,34]. A series of pyrazolopyrimidines or several botanical folk medicines have been also recently demonstrated to be KCNQ channel modulators [35,36]. KCNQ2/3 has been previously shown to be functionally active in different types of pituitary cells [15,29,37,38,39]. As such, whether and how the QO-58 can modulate the magnitude, gating, and voltage-dependent hysteresis (V_hys_) of *I*_K(M)_ residing in different types of electrically excitable cells are worthy of being further investigated.

The big-, high-, or large-conductance Ca^2+^-activated K^+^ (BK_Ca_ or BK) channels (K_Ca_1.1, KCNMA1, *Slo1*) belong to a family of voltage-activated K^+^ channels, and their activity can be increased by the elevation of intracellular Ca^2+^, membrane depolarization, or both. Due to its high conductance, the BK_Ca_ channel is hence regarded as a maxi- or large-K^+^ channel. The activity of these channels is abundantly and functionally distributed in an array of excitable and non-excitable cells. The regulation of such activity can play essential roles in various physiological or pathophysiological events, such as membrane excitability, neurotransmitter release, stimulus-secretion coupling, muscle relaxation, and pain sensation [40,41,42,43,44,45,46]. Moreover, some small molecules, such as BMS-204352, naringenin and QO-40, have been reported to activate *I*_K(M)_ as well as to enhance the activity of BK_Ca_ channels in excitable cells [9,47,48].

The *erg* (*ether-à-go-go* related gene)-mediated K^+^ current (*I*_K(erg)_), the components for which are encoded by three different subfamilies of the KCNH gene, is able to generate the pore-forming α-subunit of *erg*-mediated K^+^ (i.e., K_erg_ or K_V_11) channels [49]. The *I*_K(erg)_ is intrinsically present in different types of excitable cells. It can be engaged in the maintenance of resting potential and in modifications of the subthreshold excitability [50,51]. Whether the presence of QO-58 changes the magnitude of this type of K^+^ current is largely unknown.

Therefore, the overall objective of this study was to explore possible underlying mechanism of QO-58 actions on different ionic currents (e.g., *I*_K(M)_, *I*_K(Ca)_, and *I*_K(erg)_) present in excitable cells (e.g., pituitary GH_3_ somatolactotrophs). The investigations obtained in this study showcase the evidence demonstrating that QO-58 is capable of interacting with K_M_ or BK_Ca_ channels to stimulate the amplitude of *I*_K(M)_ or *I*_K(Ca)_, respectively, in a concentration-dependent manner in these cells. In addition to the activation of M-type K^+^ (K_M_, KCNQ or K_V_7) channels, QO-58-mediated activation of BK_Ca_-channel activity is likely to converge to act on the functional activities of different types of excitable cells.

## 2. Results

### 2.1. Stimulatory Effect of QO-58 on M-Type K^+^ Current (I_K(M)_) Recorded from Pituitary GH_3_ Cells

**Figure 1:**
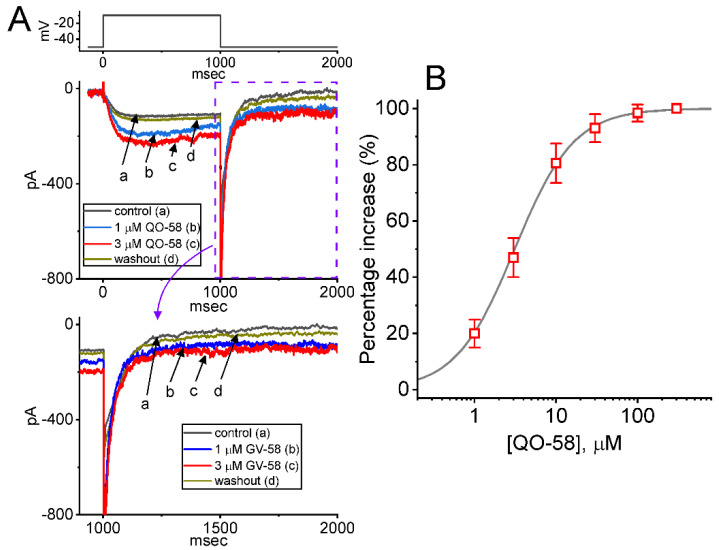
QO-58-induced stimulation of M-type K^+^ current (*I*_K(M)_) recorded from pituitary GH_3_ cells. In these experiments, we placed cells in high-K^+^ (145 mM), Ca^2+^-free solution that contained 1 μM tetrodotoxin (TTX) and 0.5 mM CdCl_2_ and the electrodes that we used were filled up with a K^+^-containing solution. (**A**) Superimposed current traces obtained in the control period (a), during cell exposure of 1 μM QO-58 (b) or 3 μM QO-58 (c), and washout of QO-58 (d). The top part indicates the voltage-clamp protocol applied, while the lower part is an expanded record from purple dash box in the upper part. (**B**) Concentration-dependent stimulation of QO-58 effect on the amplitude of *I*_K(M)_ (mean ± SEM; *n* = 8 for each point). Current amplitudes during cell exposure to different QO-58 concentrations were measured at the end of depolarizing pulse from −50 to −10 mV with a duration of 1 s. Sigmoid smooth curve indicates best fit to a modified Hill function described in Section 4.

In an initial stage of experiments, we wanted to test if the exposure to QO-58 produced any adjustments on the magnitude of I_K(M)_ identified in these cells. To amplify I_K(M)_ amplitude [31,52], we used high-K^+^ (145 mM), Ca^2+^-free solution as a bathing medium, and the recording pipette was filled up with a K^+^-containing solution. As illustrated in Figure 1, one minute after addition of QO-58 (1 or 3 μM), the I_K(M)_ magnitude evoked in response to 1-s depolarizing step from −50 to −10 mV progressively increased. For example, under cell exposure to 3 μM QO-58, I_K(M)_ amplitude was evidently increased, as demonstrated by a considerable raise in the amplitude to 201 ± 26 pA (*n* = 8, *p* < 0.05) from a control value of 118 ± 19 pA (*n* = 8). The QO-58-mediated increase in I_K(M)_ observed herein was also accompanied by the fastened activation time course of the current, as demonstrated by a shortening in the value of activation time constant (τ_act_) from 68 ± 8 to 22 ± 4 ms (*n* = 8, *p* < 0.05) during exposure to 3 μM QO-58. After washout of QO-58, current amplitude returned to 122 ± 21 pA (*n* = 8, *p* < 0.05). Moreover, QO-58-mediated stimulation of I_K(M)_ was attenuated by further addition of thyrotropin releasing hormone (TRH, 1 μM) or linopirdine (10 μM), but not by iberiotoxin (200 nM). The results were demonstrated by a decrease of current amplitude during further exposure to 1 μM TRH or 10 μM linopirdine to 125 ± 21 pA (*n* = 8, *p* < 0.05) or 127 ± 21 pA (*n* = 8, *p* < 0.05), respectively. TRH or linopirdine was reported to suppress I_K(M)_ effectively in pituitary lactotrophs [18,31,49], while iberiotoxin is known to block large-conductance Ca^2+^-activated K^+^ (BK_Ca_) channels.

The relationship between the QO-58 concentration and the percentage increase of I_K(M)_ was ascertained and is hence illustrated in Figure 1B. To evoke I_K(M)_ obtained in the control period (i.e., absence of QO-58) and during cell exposure to different concentrations (1–300 μM) of QO-58, each cell was depolarized from −50 to −10 mV with a duration of 1 s. Addition of QO-58 was noticed to increase the amplitude of I_K(M)_ in a concentration-dependent fashion. As the data became least-squares fitted to a Hill function as stated in Section 4, the half-maximal concentration (i.e., EC_50_) required for stimulatory effect of QO-58 on I_K(M)_ was calculated to be 3.1 μM. The data from this set of experiments reflect that QO-58 has a stimulatory effect on I_K(M)_ in GH_3_ cells in a concentration-dependent manner.

### 2.2. Effect of QO-58 on Average Current Versus Voltage (I-V) Relationship and Steady-State Activation Curve of I_K(M)_

**Figure 2:**
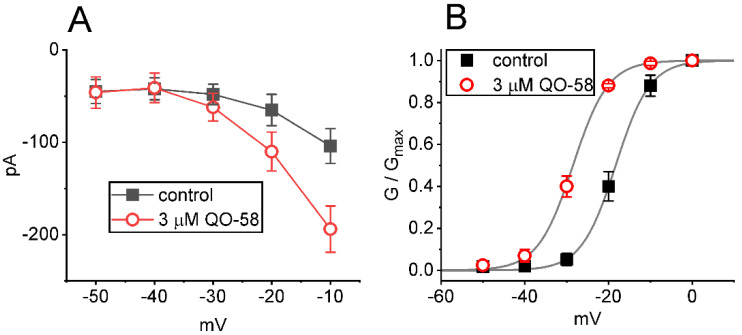
Effect of QO-58 on the current versus voltage (*I-V*) relationship (**A**) and the activation curve (**B**) of *I*_K(M)_. The experimental protocol applied is the same as that used in Figure 1. (**A**) Average *I-V* relationship of *I*_K(M)_ amplitude acquired in the control period (i.e., absence of QO-58, solid black squares) and during cell exposure to 3 μM QO-58 (open red circles). (**B**) Steady-state activation curve of *I*_K(M)_ in the absence (solid black squares) and presence (open red circles) of 3 μM QO-58 (mean ± SEM; *n* = 8). Smooth curves drawn were optimally fitted to a modified Boltzmann function as elaborated in Section 4. Of note, there is a leftward shift along the voltage axis in the quasi-steady-state activation curve of *I*_K(M)_ in QO-58 presence, despite being void of changes in the gating charge of the curve.

We next continued to study if the presence of QO-58 can modify the *I*_K(M)_ amplitude measured at different levels of membrane potentials. The average *I-V* relationship of *I*_K(M)_ with or without the QO-58 application is illustrated in Figure 2A. The current amplitude noticeably arose as the membrane potential became depolarized to −30 mV, and the magnitude of QO-58-stimulated *I*_K(M)_ at the level of −10 mV was higher than that at −20 or −30 mV. The relationship of relative *I*_K(M)_ conductance versus membrane potential acquired in the control period (i.e., QO-58 was not present) and during cell exposure to 3 μM QO-58 was constructed (Figure 2B). The continuous sigmoidal curve derived from experimental data sets was optimally fitted with a modified Boltzmann function (described in Section 4). In control, *V_1/2_* = −18.3 ± 0.7 mV (*n* = 8), *q* = 6.2 ± 0.2 *e* (*n* = 8), and in the presence of 3 μM QO-58, *V_1/2_* = −28.5 ± 0.8 mV (*n* = 8), *q* = 6.1 ± 0.2 *e* (*n* = 8). The results enabled us to reflect that in addition to increasing *I*_K(M)_ conductance, the presence of QO-58 could exert a leftward shift (approximately 10 mV) along the voltage axis in the activation curve of the current, albeit with no change in the gating charge of the activation curve.

### 2.3. Effect of QO-58 on I_K(M)_ Triggered by Triangular Ramp Pulse (V_ramp_) with Varying Durations

**Figure 3:**
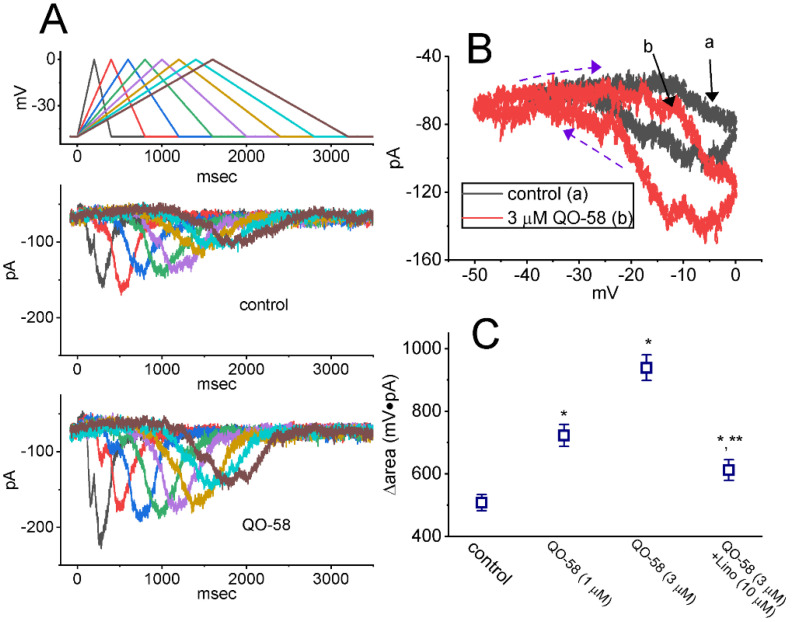
Effect of QO-58 on *I*_K(M)_ in response to an upright isosceles-triangular ramp pulse (V_ramp_) observed in GH_3_ cells. The V_ramp_ with different durations (0.4–3.2 s) (or with ramp speed between ±31.25 and ±250 mV/s) was designed to mimic different depolarizing and repolarizing slopes in bursting patterns of neuronal firing. (**A**) Representative *I*_K(M)_ traces in response to the double-pulse V_ramp_ in the absence (upper) and presence (lower) of 3 μM QO-58. The voltage protocol used is indicated in the uppermost part, and voltage waveforms appearing in different colors are indicated to correspond with current traces evoked by the waveforms. (**B**) Effect of QO-58 (3 μM) on voltage-dependent hysteresis (V_hys_) (i.e., the relationship of forward and backward current versus membrane voltage) of *I*_K(M)_ evoked by triangular V_ramp_ with a duration of 3.2 s. Black or red current trajectory indicates the absence or presence of 3 μM QO-58, respectively. The direction of *I*_K(M)_ in which time passes during the elicitation by 3.2 s triangular V_ramp_ is indicated by dashed arrows. (**C**) Summary scatter graph showing the effect of QO-58 on the V_hys_’s ∆area (mean ± SEM; *n* = 8 for each point). ∆area means the area encircling the forward (upsloping) and backward (downsloping) limbs of current trajectory evoked by V_ramp_. * Significantly different from control (*p* < 0.05), and ** significantly different from 3 μM QO-58 alone group (*p* < 0.05).

Earlier reports have shown the capability of *I*_K(M)_ strength to modulate the patterns of bursting firing in central neurons [13,15,16,17,20]. Therefore, we continued to evaluate how the presence of QO-58 could have any perturbations on *I*_K(M)_ responding to upright triangular V_ramp_ with varying durations. These V_ramp_ waveforms were specifically designed and, during the measurements, thereafter, delivered to the tested cell through digital-to-analog conversion. In the current experimental scenario, we voltage-clamped the cell at −50 mV, and the upsloping (forward) limb from −50 to 0 mV followed by the downsloping (reverse) limb back to −50 mV with varying duration (0.4–3.2 s) was imposed over it. As demonstrated in Figure 3, the peak amplitude of *I*_K(M)_ became progressively declined with increasing the V_ramp_’s duration (or decreasing the V_ramp_’s speed); however, as the V_ramp_’s slope decreased, the maximal strength of *I*_K(M)_ triggered by the upsloping limb of triangular V_ramp_ progressively increased. Moreover, it can be seen that under cell exposure to 3 μM QO-58, the current magnitude responding to both rising and falling V_ramp_ was increased (Figure 3A,B). For example, as the during of triangular V_ramp_ was set at 3.2 s (i.e., slope = 31.25 mV/s), the application of 3 μM QO-58 increased the current amplitude measured from the upsloping or downsloping limb at the level of −10 mV from 63 ± 4 to 81 ± 5 pA (*n* = 8, *p* < 0.05) or from 91 ± 6 to 148 ± 11 pA (*n* = 8, *p* < 0.05), respectively. The experimental observations project that the strength of *I*_K(M)_ in the upsloping limb was obviously increased as the duration of triangular V_ramp_ increased, while that in the downsloping end progressively decreased, and that the presence of QO-58 contributed to an increase in *I*_K(M)_ in a time- and state-dependent manner in GH_3_ cells.

The voltage-dependent hysteresis (V_hys_) of ionic currents have been growingly noticed to exert important impacts on electrical behaviors of action potential firing [29,31,53,54]. As illustrated in Figure 3A,B, the *I*_K(M)_ amplitude triggered by the upsloping limb of upright triangular V_ramp_ was evidently lower than that by the downsloping end, strongly reflecting that a V_ramp_-induced V_hys_ behavior resides in *I*_K(M)_ observed in GH_3_ cells. As the duration of triangular V_ramp_ increased from 0.4 to 3.2 s (i.e., the V_ramp_’s slope became decreased), the V_hys_ degree was reduced. Of notice, by adding QO-58 (3 μM), *I*_K(M)_ evoked during the upsloping limb of triangular V_ramp_ arose to a less extent than that measured at the downsloping ramp. For example, in control period (i.e., absence of QO-58), *I*_K(M)_ at the level of −15 mV during the upsloping and downsloping ends of triangular V_ramp_ were 53 ± 6 pA (*n* = 8) and 91 ± 8 pA (*n* = 8), respectively, the values between which were noticed to differ significantly (*p* < 0.05). Moreover, by adding QO-58 (3 μM), the amplitudes of forward and backward *I*_K(M)_ at the same level of voltage noticeably increased to 69 ± 7 pA (*n* = 8, *p* < 0.05) and 138 ± 11 pA (*n* = 8, *p* < 0.05), respectively. Therefore, the magnitude of QO-58-mediated current stimulation at the upsloping (forward) and downsloping (backward) limbs of triangular V_ramp_ differ significantly. The presence of 3 μM QO-58 increased *I*_K(M)_ amplitude at −15 mV during the upsloping or downsloping limb of triangular V_ramp_ by about 16% or 52%, respectively.

We further quantified the degree (i.e., V_hys_’s ∆area) of V_ramp_-induced V_hys_ of *I*_K(M)_. The results demonstrated that the amount of V_hys_ responding to 3.2 s triangular V_ramp_ was considerably increased in the presence of QO-58. Figure 3C summarizes the data demonstrating the effects of QO-58 (1 or 3 μM) on the area encircling the forward and backward current trajectory of V_ramp_-evoked *I*_K(M)_. For example, in addition to its stimulation of *I*_K(M)_ amplitude, cell exposure of 3 μM QO-58 resulted in an increase in the V_hys_ strength responding to long-lasting triangular V_ramp_, as illustrated by a considerable increase in V_hys_’s ∆area arising from 508 ± 26 to 939 ± 41 mV·pA (*n* = 8, *p* < 0.05). Moreover, during the continued exposure to 3 μM QO-58, subsequent exposure to 10 μM linopirdine appreciably attenuated the ∆area to 612 ± 33 mV·pA (*n* = 8, *p* < 0.05). Linopirdine was reported to be a blocker of K_M_ channels [18]. It is plausible to assume, therefore, that QO-58 is effective in stimulating *I*_K(M)_ residing in GH_3_ cells in a V_hys_-dependent manner.

### 2.4. Effect of QO-58 on M-Type K^+^ Channel (K_M_) Channels Measured from GH_3_ Cells

**Figure 4:**
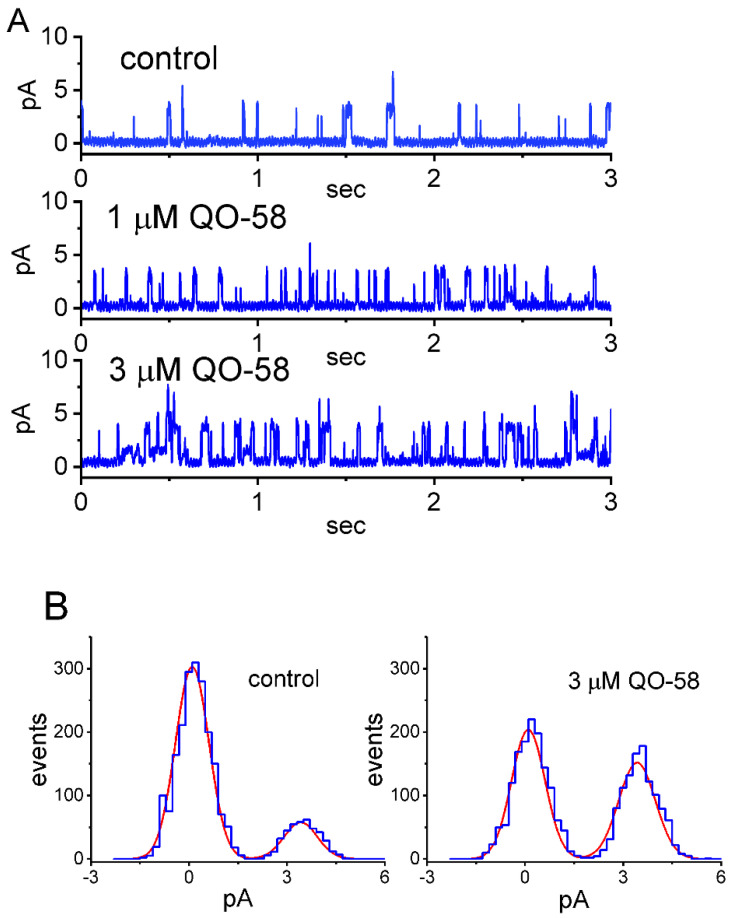

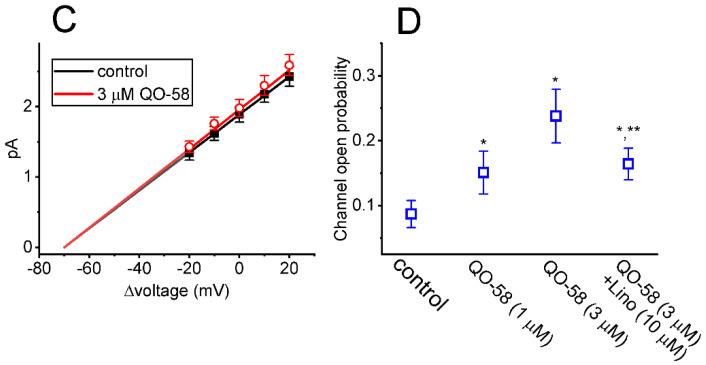
Stimulatory effect of QO-58 on the activity of M-type K^+^ (K_M_) channels recorded from GH_3_ cells. In this series of cell-attached current recordings, cells were bathed in high-K^+^, Ca^2+^-free solution, and we filled up the recording pipette with low-K^+^ (5.4 mM) solution. (**A**) Representative single K_M_ channels in the control period (upper) and during cell exposure to 1 μM QO-58 (middle) or 3 μM QO-58 (lower). The channel activity in the absence or presence of QO-58 was taken at the level of +20 mV relative to the bath. The upward deflection indicates the opening event of the channel. (**B**) Amplitude histogram taken in the absence (left) and presence (right) of 3 mM QO-58. (**C**) Average *I-V* relationship of single K_M_ channels in the absence (solid black squares) and presence (open red circles) of 3 μM QO-58 (mean ± SEM; *n* = 8 for each point). Of note, the linear relationship of K_M_ channels versus ∆voltage was superimposable between the absence and presence of QO-58; hence, the single-channel conductance of the channel between the absence and presence of 3 μM QO-58 did not differ. (**D**) Summary scatter graph showing effect of QO-58 (1 and 3 μM) and QO-58 (3 μM) plus linopirdine (Lino, 10 μM) on the probability of K_M_ channels that would be open (mean ± SEM; *n* = 8 for each point). Channel activity was measured at +20 mV relative to the bath. * Significantly different from control (*p* < 0.05) and ** significantly different from QO-58 (3 μM) alone group (*p* < 0.05).

The QO-58-stimulated whole-cell *I*_K(M)_ detected above in these cells could arise from changes occurring in either channel open probability, single-channel amplitude, gating kinetics, or the number of K_M_ channel. The reasons therefore enabled us to investigate the single-channel recordings of the channel with or without the presence of QO-58. In this set of cell-attached current recordings, we placed cells in high-K^+^, Ca^2+^-free solution, and the recording pipette was filled with low-K^+^ (5.4 mM) solution. As demonstrated in Figure 4A, when the examined cell was maintained at +20 mV relative to the bath, the activity of single K_M_ channel was robustly observed [31,55]. Of particular interest, as QO-58 was applied to the bath, the probabilities of K_M_-channel openings progressively increased. For example, the presence of 3 μM QO-58 significantly increased the channel open probability from 0.087 ± 0.021 to 0.238 ± 0.041 (*n* = 8, *p* < 0.05); however, there was devoid of changes in single-channel amplitude (Figure 4B). Moreover, in continued presence of 3 μM QO-58, linopirdine (10 μM) resulted in an attenuation of QO-58-stimulated channel activity, as demonstrated in a significant reduction in channel activity to 0.164 ± 0.025 (*n* = 8, *p* < 0.05).

### 2.5. Effect of QO-58 on the Single-Channel Conductance and Activation Curve of K_M_ Channels

We further examined if K_M_-channel activity measured at different levels of membrane potentials could be altered by the presence of QO-58. As demonstrated in Figure 4C, the single channel conductance of K_M_ channels achieved with or without application of QO-58 did not significantly differ (27 ± 3 pS [in control] versus 28 ± 3 pS [in the presence of 3 μM QO-58]; *n* = 8, *p* > 0.05), despite the increased probability of K_M_-channel openings in its presence. A summary showing effects of QO-58 and QO-58 plus linopirdine on K_M_-channel activity in GH_3_ cells is also presented in Figure 4D. The results led us to reflect that as GH_3_ cells were continually exposed to QO-58 (3 μM), the channel open probability was significantly attenuated by subsequent addition of linopirdine (10 μM).

### 2.6. QO-58-Mediated Stimulation of Ca^2+^-Activated K^+^ Currents (I_K(Ca)_) by the Presence of QO-58

**Figure 5:**
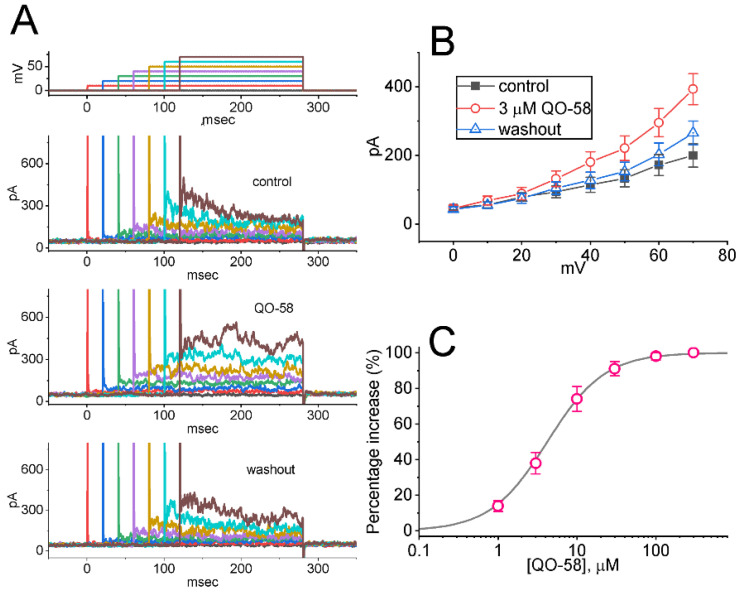
Stimulatory effect of QO-58 on the amplitude of whole-cell (i.e., macroscopic) Ca^2+^-activated K^+^ current (*I*_K(Ca)_) measured from GH_3_ cells. In this series of voltage-clamp current recordings on these cells, we used normal Tyrode’s solution containing 1.8 mM CaCl_2_ as a bathing medium, and the recording pipette used was backfilled with K^+^-enriched solution. As whole-cell configuration was established, we evoked *I*_K(Ca)_ from a holding potential of 0 mV to test potentials in the range of 0 and +70 mV (10-mV in increments) at a rate of 0.1 Hz. (**A**) Superimposed current traces activated in response to a series of voltage steps (indicated in the uppermost part). Current traces in the upper part are controls (i.e., absence of QO-58), those in the middle part were recorded during cell exposure to 3 μM QO-58, while those in lower part were taken after washout of the QO-58. The duration of rectangular voltage commands applied was set in the range of 280 and 160 ms (20-ms decrements), indicating a progressive increase with membrane depolarization (i.e., an outwardly-rectifying property). (**B**) Average *I-V* relationship of *I*_K(Ca)_ obtained in the control period, during the exposure to 3 μM QO-58, and after washout of QO-58. Current amplitude was measured at the end of each depolarizing pulse. Each point represents the mean ± SEM (*n* = 8). (**C**) Concentration-response relationship for QO-58-induced stimulation of *I*_K(Ca)_ (mean ± SEM; *n* = 8 for each point). The gray continuous line drawn is reliably fitted to the Hill equation. The values for EC_50_ or n_H_ were yielded to be 4.2 μM or 1.2, respectively.

Several small molecules (e.g., BMS-204352, naringenin, and QO-40) that demonstrated to stimulate K_M_-channel activity have been reportedly noted to regulate other types of K^+^ currents (e.g., *I*_K(Ca)_) [9,47,48]. For these reasons, we next explored if QO-58 is able to modify the amplitude of *I*_K(Ca)_ residing in GH_3_ cells. In these experiments we voltage-clamped the tested cell at a holding potential of 0 mV to prevent the interference by other type of ionic currents (i.e., voltage-gated Ca^2+^ currents) [56,57]. As demonstrated in Figure 5A, one minute after cell exposure to 3 μM QO-58, *I*_K(Ca)_ amplitudes measured at different levels of membrane potentials increased. Average *I-V* relationships of *I*_K(Ca)_ with or without the QO-58 (3 μM) presence are illustrated in Figure 5B. The concentration-dependent stimulation by QO-58 of macroscopic *I*_K(Ca)_ amplitude was established and is hence depicted in Figure 5C. According to the Hill equation described in Section 4, the EC_50_ value required for QO-58-stimulated effect on *I*_K(Ca)_ was calculated to be 4.2 μM.

### 2.7. Stimulatory Effect of QO-58 on the Activity of Large-Conductance Ca^2+^-Activated K^+^ (BK_Ca_) Channels Identified in GH_3_ Cells

**Figure 6:**
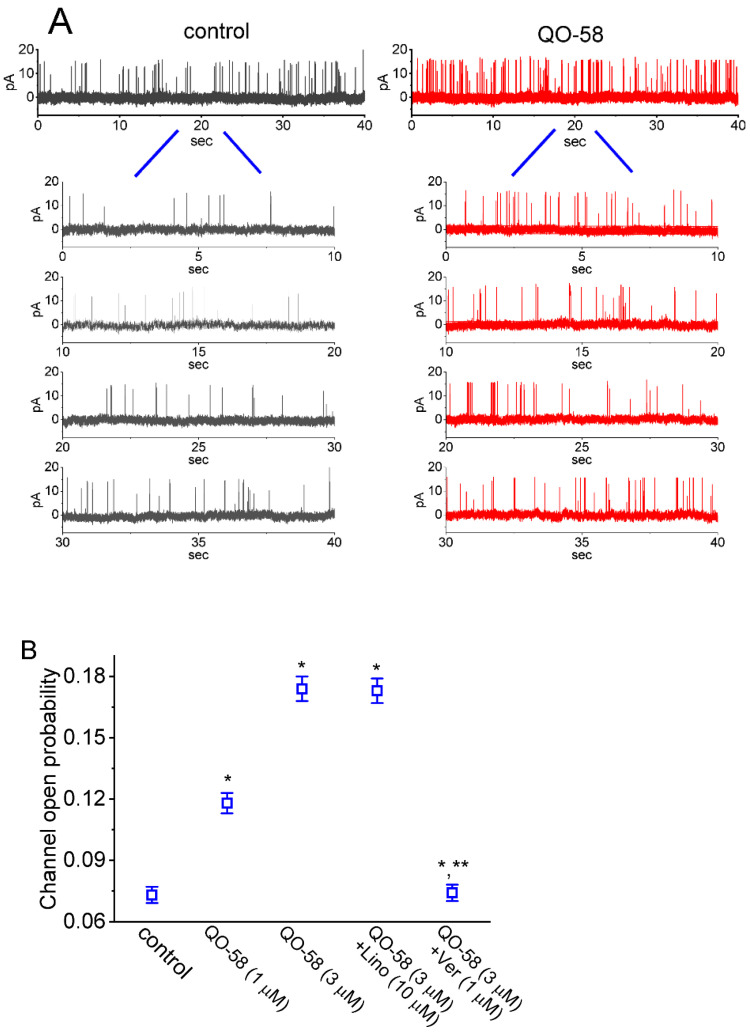
Stimulatory effect of QO-58 on the activity of BK_Ca_ channels recorded from GH_3_ cells. We conducted these inside-out current recordings in cells which were bathed in high-K^+^ solution containing 0.1 μM Ca^2+^, and the recording pipette was then filled up with K^+^-containing solution. (**A**) Original BK_Ca_-channel currents obtained in the control period (left, black color) and after bath application of 3 μM QO-58 (right, red color). The detached patch was voltage-clamped at +60 mV. The lower part indicates the expanded records from the uppermost part. The opening event of the channel is indicated by the upward deflection. (**B**) Summary scatter graph showing effect of QO-58, QO-58 plus linopirdine (Lino), or QO-58 plus verruculogen (Ver) (mean ± SEM; *n* = 8 for each point). Under inside-out configuration, the channel open probability was measured at the level of +60 mV. * Significantly different from control (*p* < 0.05) and ** significantly different from QO-58 (3 μM) alone group (*p* < 0.05).

The QO-58-induced raise in whole-cell *I*_K(Ca)_ described above could be mediated through either adjustment in channel open probability, single-channel amplitude, gating kinetics of the BK_Ca_ channels, or in any combinations. Therefore, these reasons urged us to assess the single-channel activities of the channels functionally active in GH_3_ cells. In this set of inside-out current recordings, we bathed the tested cells in high-K^+^ solution containing 0.1 μM Ca^2+^, and the recording pipette was filled up with K^+^-containing solution. As demonstrated in Figure 6A, as the excised patch was voltage-clamped at +60 mV, the activity of BK_Ca_ channels occurring in rapid and independent open-closed transitions was robustly detected. One minute after bath addition of QO-58, the channel opening probability was conceivably increased. For example, under inside-out configuration, QO-58 at a concentration of 1 or 3 μM applied to bath medium led to a respective increase in channel opening probability to 0.118 ± 0.005 (*n* = 8, *p* < 0.05) or 0.174 ± 0.006 (*n* = 8, *p* < 0.05) from a control value of 0.073 ± 0.004 (*n* = 8). However, single-channel amplitude of BK_Ca_ channel was not noticed to differ significantly between the absence and presence of 3 μM QO-58 (12.8 ± 2 pA [in control] versus 12.9 ± 2 pA [in the presence of 3 μM QO-58]; *n* = 8, *p* > 0.05). Under the exposure to 3 μM QO-58, the slow component of mean closed time of the channel became considerably shortened to 19 ± 3 ms (*n* = 8, *p* < 0.05) from a control value of 32 ± 5 ms (*n* = 8). Of additional notice, as the excised patch was continually exposed to 3 μM QO-58, subsequent addition of verruculogen (1 μM) considerably decreased channel open probability to 0.074 ± 0.004 (*n* = 8, *p* < 0.05), although further application of linopirdine (10 μM) produced minimal effect on it (0.174 ± 0.006 [in the presence of 3 μM QO-58 alone] versus 0.173 ± 0.006 [in presence of QO-58 plus linopirdine]; *n* = 8, *p* > 0.05) (Figure 6B). Verruculogen is a tremorgenic mycotoxin known to effectively suppress the activity of BK_Ca_ channels [58,59]. Therefore, the experimental results strongly indicate that QO-58-activated channel activity is mainly through its activation of BK_Ca_ channels, rather than that of K_M_ channels. They also enable us to project that the activation is attributed primarily to the shortening of mean closed time of the channel, despite no change in single-channel amplitude in the presence of QO-58.

**Figure 7:**
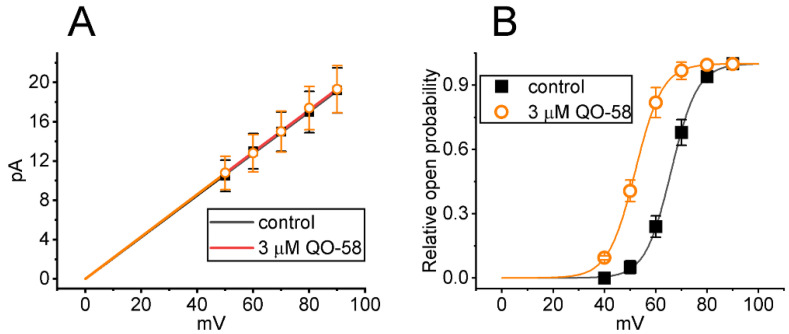
Effect of QO-58 on single-channel conductance (**A**) and activation curve (**B**) of BK_Ca_ channels in GH_3_ cells. In this series of inside-out current recordings, we voltage-clamped the excised patched at different levels of membrane potentials, and the recording pipette was filled with K^+^-enriched solution. (**A**) Average *I-V* relationship of single BK_Ca_-channel currents (i.e., linear regression between membrane potential and mean single-channel amplitude) obtained in the absence (black filled squares) and presence (orange open circles) of 3 μM QO-58 (mean ± SEM; *n* = 8 for each point). Of note, the linear relationship of BK_Ca_ channels between the absence and presence of QO-58 is superimposed, and the value of reversal potential with or without the QO-58 addition was pointed toward zero mV. (**B**) Steady-state activation curve of BK_Ca_ channels obtained in the control period (black filled squares) and during exposure to 3 μM QO-58 (orange open circles) (mean ± SEM; *n* = 8 for each point). Sigmoid lines indicate the best fit to a Boltzmann function as stated in Section 4. Of note, there is a leftward shift of the activation curve of the channel, although neither single-channel conductance of the channel nor gating charge of the curve was altered by QO-58 presence.

We further explored how the presence of QO-58 alters BK_Ca_-channel activity at different levels of membrane potentials. As demonstrated in Figure 7A, the linear relationship of single-channel amplitude versus membrane potential (i.e., single-channel conductance) was collated under inside-out configuration. As the excised patch was exposed to 3 μM QO-58, the value of single-channel conductance obtained between the absence and presence of QO-58 did not differ significantly (213 ± 8 pS [in control] versus 215 ± 9 pS [in the presence of QO-58]; *n* = 8, *p* > 0.05). Additionally, the steady-state activation curve of BK_Ca_ channels with or with the QO-58 application is illustrated in Figure 7B. As the smooth lines drawn by fitting the data to the Boltzmann equation stated in Section 4, the results demonstrated that during the exposure to QO-58, there was a leftward shift (approximately 14 mV) along the voltage axis in the activation curve of the channel with no appreciable modifications in gating charge. For example, in control, *V_1/2_* = +66 ± 8 mV and *q* = 4.9 ± 0.2 *e* (*n* = 8), while in the presence of 3 μM QO-58, *V_1/2_* = +52 ± 7 mV and *q* = 4.8 ± 0.2 *e* (*n* = 8). These results indicated that although neither single-channel conductance nor gating charge of the channel was changed, the steady-state activation curve of BK_Ca_ channels measured under inside-out excised patch of GH_3_ cells was shifted toward less depolarized potential, as the detached patch was exposed to QO-58.

### 2.8. Minor Inhibitory Effect of QO-58 on Erg-Mediated K^+^ Current (I_K(erg)_) Seen in GH_3_ Cells

**Figure 8:**
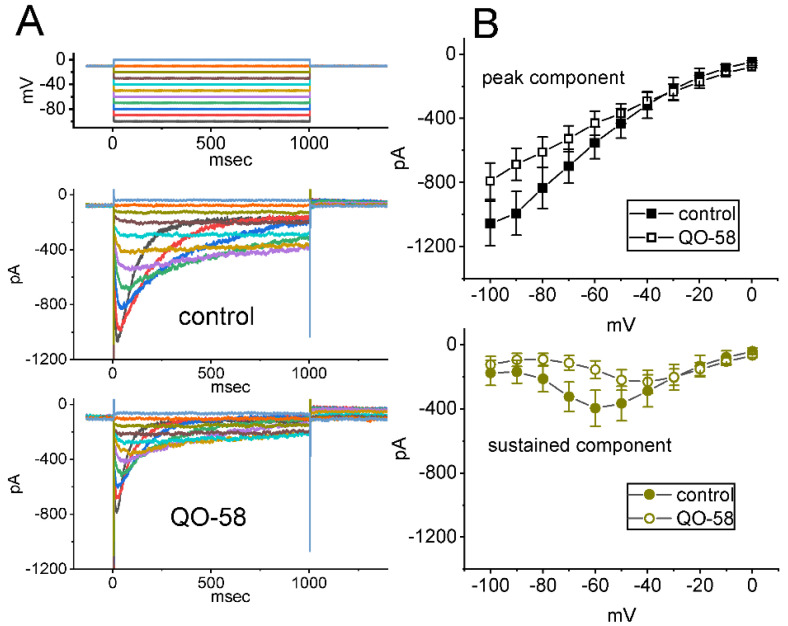
Minor inhibition of *erg*-mediated K^+^ current (*I*_K(erg)_) produced by the presence of QO-58 in GH_3_ cells. In these experiments, we used high-K^+^, Ca^2+^-free solution as bathing medium and the recording pipette was filled up with K^+^-containing (145 mM) solution. (**A**) Superimposed current traces obtained in the control period (upper) and during cell exposure to 10 μM QO-58 (lower). The top part indicates the voltage-clamp protocol imposed. (**B**) Average *I-V* relationship of peak (black squares, upper) or sustained (brown circles, lower) component of *I*_K(erg)_ obtained in the absence (filled symbols) and presence (open symbols) of 10 μM QO-58 (mean ± SEM; *n* = 7 for each point). Peak or sustained *I*_K(erg)_ obtained with or without the QO-58 addition was measured at the start or end-point of each hyperpolarizing step with a duration of 1 s. Of note, the presence of 10 μM QO-58 slightly inhibited *I*_K(erg)_ in these cells.

In another set of experiments, we attempted to explore if the presence of QO-58 could have any influence on another type of whole-cell K^+^ current (i.e., *I*_K(erg)_). The measurements were conducted in these cells bathed in high-K^+^, Ca^2+^-free solution containing 1 μM TTX and 0.5 mM CdCl_2_, and the pipette that was used was filled up with K^+^-enriched solution. The tested cell was voltage-clamped at −10 mV, and a series of command voltage steps ranging between −100 and 0 mV was thereafter delivered to evoke deactivating *I*_K(erg)_ [12,37,52,60,61,62,63]. As illustrated in Figure 8, under cell exposure to 10 μM QO-58, average *I-V* relationship of *I*_K(erg)_ became lessened in the voltages ranging between −100 and −50 mV. For example, at the level of −100 mV, the exposure to 10 μM QO-58 significantly reduced the peak amplitude of hyperpolarization-activated *I*_K(erg)_ by 25 ± 2% from 1055 ± 139 to 791 ± 113 pA (*n* = 7, *p* < 0.05). After the compound’s washout, current amplitude returned to 1012 ± 131 pA (*n* = 7, *p* < 0.05). Therefore, it is noticeable that unlike QO-58 effect on *I*_K(M)_ or *I*_K(Ca)_, *I*_K(erg)_ functionally active in GH_3_ cells is susceptible to minor inhibition by the QO-58 presence.

### 2.9. Docking Results on Interaction between K_Ca_1.1 Channel and QO-58 or between KCNQ2 and QO-58

**Figure 9:**
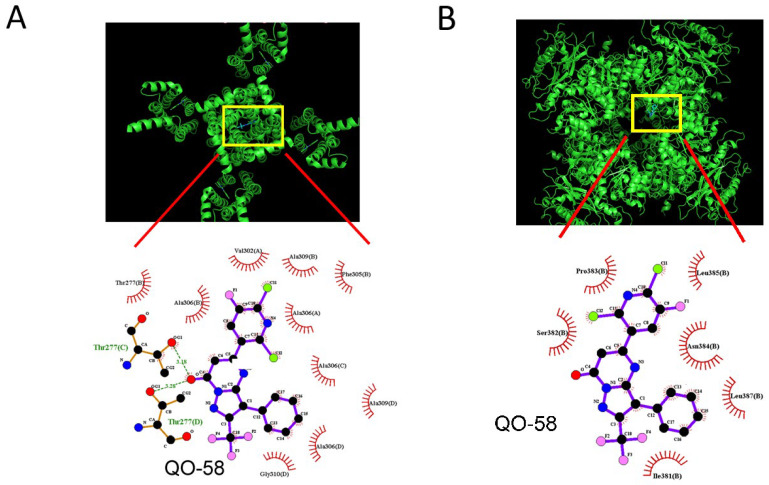
Docking results of K_Ca_1.1 channel and QO-58 (**A**) or KCNQ2 and QO-58 (**B**). Protein structure of K_Ca_1.1 or KCNQ2 channel was acquired from PDB (PDB ID: 6V3G or 7CR1), respectively, while chemical structure of QO-58 was from PubChem (Compound CID: 51351551). The structure of K_Ca_1.1 or KCNQ2 channel was docked by the QO-58 molecule through PyRx (https://pyrx.sourceforge.io/, accessed on 23 June 2022). The diagram of interactions between K_Ca_1.1 or KCNQ2 channel and the QO-58 molecule was generated by LigPlot^+^ (https://www.ebi.ac.uk/thornton-srv/software/LIGPLOT/, accessed on 23 June 2022). Of note, the red arcs with spokes radiating toward the ligand (i.e., QO-58) in the lower part of each panel denote the hydrophobic contact, while green dot line indicates the hydrogen bond.

In a final set of studies, we investigated how the protein of K_Ca_1.1 (or α-subunit of BK_Ca_ channel) or KCNQ2 could be docked with QO-58 by exploiting PyRx software. The predicted binding sites of the QO-58 molecule were demonstrated in Figure 9. Of notice, with being docked to K_Ca_1.1 with a binding energy of −7.1 kcal/mol, QO-58 can form hydrogen bond with residue Thr 277, while it is able to have hydrophobic contact with residue Thr 277, Val 302, Phe 305, Ala 306, Ala 309, and Gly 310. Alternatively, as being docked to KCNQ2 with a binding energy of −6.9 kcal/mol, QO-58 can form hydrophobic contact with residue Ile 381, Ser 382, Pro 383, Asn 384, Leu 385, and Leu 387. Of notice, these results thus prompted us to reflect that KCNQ2 and BK_Ca_ channels are likely to share unique motifs or recognition sequences with which QO-58 or other structurally similar compounds can interact, and that QO-58 can bind to the cytoplasmic residues of KCNQ2 or K_Ca_1.1 channel, which are adjacent to transmembrane segment of the channel. Therefore, care needs to be mentioned in attributing the actions of QO-58 or other structurally similar compounds exclusively to the stimulation of KCNQx- (K_V_7-) channel activity as reported previously [4,8,18].

## 3. Discussion

The noticeable conclusions are drawn from this study as follows. First, the presence of QO-58 concentration-dependently increased the amplitude of *I*_K(M)_ and *I*_K(Ca)_ with EC_50_ value of 3.1 and 4.2 μM, respectively. Second, under cell exposure to QO-58, the steady-state activation curve of *I*_K(M)_ was shifted along voltage axis to a hyperpolarized potential with no change in the gating charge. Third, the V_hys_ strength of *I*_K(M)_ activated by triangular V_ramp_ measurably increased by the QO-58 presence. Fourth, cell exposure to QO-58 enhanced the probability of BK_Ca_-channel openings as well as shifted the activation curve of the channel at steady state toward the less depolarized potential; however, neither the gating charge nor single-channel conductance of the channel was affected during its exposure. Fifth, the deactivating *I*_K(erg)_ activated by membrane hyperpolarization was slightly suppressed by adding QO-58. Taken together, the interaction of QO-58 with K_M_ or BK_Ca_ channels to stimulate *I*_K(M)_ or *I*_K(Ca)_ in excitable cells is expected to occur in a concentration- and voltage-dependent manner, assuming that similar in vivo findings occur.

In agreement with previous studies [2], the current observations demonstrated that with optimum EC_50_ of 3.1 μM, QO-58 was capable of enhancing the magnitude of *I*_K(M)_ seen in GH_3_ cells. Furthermore, the V_hys_ changes have been regarded to play an essential characteristic in electrical behaviors of different excitable cells. In the current study, in accordance with earlier studies [29,31,53,54], the *I*_K(M)_ intrinsically residing in GH_3_ cells was robustly observed to undergo V_ramp_-induced V_hys_, suggesting that the voltage sensitivity of gating charge movements is dependent on the previous state (or conformation) of the K_M_ channel. In other words, as the membrane potential becomes depolarized (i.e., during initiation of action potential or the upsloping limb of the triangular V_ramp_), the voltage dependence of *I*_K(M)_ activation would switch to less depolarized voltages with a small current magnitude, thereby have the tendency to decrease cell excitability. However, as the membrane potential becomes negative (i.e., downward ramp of the double V_ramp_), the voltage dependence of K_M_ channels may shift the mode of V_hys_ to one which occurs at more negative potentials, thereby leading to an increase in membrane repolarization. Furthermore, upon triangular V_ramp_ with varying durations, the QO-58 addition noticeably increased the V_hys_’s strength for *I*_K(M)_ elicitation. Under this scenario, we extended previous results and further provided the experimental observations, strongly indicating that there would be a perturbing stimulatory effect of QO-58 on such non-equilibrium property (i.e., V_hys_) in K_M_ (or K_V_7) channels in electrically excitable cells. However, how QO-58-induced changes in *I*_K(M)_’s V_hys_ are linked to the behavior of these cells occurring in vivo remains unclear. Of importance, the main point raised is that the adjustments by QO-58 of *I*_K(M)_’s V_hys_ residing in excitable cells are anticipated to be responsible for altering the bursting pattern of action potentials in excitable cells [13,14,15,16,17,19,20,30,60].

In the present observations, effective EC_50_ value needed for QO-58-stimulated *I*_K(Ca)_ present in GH_3_ cells was yielded to be 4.2 μM, a value that is close to that (3.1 μM) for its activation of *I*_K(M)_. Under our inside-out current recordings, the addition of QO-58 to bath medium was able to increase the probability of BK_Ca_-channel openings with being void of change in single-channel conductance, suggesting that QO-58 may bind to a site located on the cytoplasmic leaflet of the α-subunit of BK_Ca_ channels. The slow component of mean closed time of the channel decreased by adding this compound. Under its exposure, the steady-state activation curve of BK_Ca_ channels seen in GH_3_ cells became overly shifted to less depolarized potential with no appreciable change in the gating charge of the curve. The QO-58-stimulated BK_Ca_ channel activity was also effectively counteracted by subsequent addition of verruculogen (1 μM), yet not by linopirdine (10 μM). Verruculogen is known to block BK_Ca_ channels effectively [58,59], while linopirdine can suppress K_M_-channel activity [18,31,33]. Under such scenario, it is plausible to notice that apart from its effects on *I*_K(M)_, QO-58-stimulated *I*_K(Ca)_ arises primarily through the observed activation of BK_Ca_ channels, although the precise or detailed ionic mechanism of QO-58 actions on the activity and gating kinetics of BK_Ca_ channels remains to be resolved.

According to previous pharmacokinetic studies, peak plasma concentration after the oral administration with QO58-lysine with a dose of 50, 20, 12.5 mg/kg has been reported to reach around 50 μg/mL (85 μM), 20 μg/mL (34 μM), or 4 μg/mL (6.8 μM), respectively [5,7]. Moreover, the sensitivity of voltage-clamped cells (e.g., neuroendocrine or endocrine cells) to QO-58 or other structurally similar compounds (e.g., QO-40) can be expected to depend not only on the QO-58 concentration applied, but also greatly on different confounding variables which include the pre-existing level of resting potential and varying bursting patterns of action potential firing. It is therefore conceivable to reflect that QO-58-mediated concerted stimulation of K_M_ (KCNQx or K_V_7x) and BK_Ca_ channels seen in GH_3_ cells is of pharmacological or therapeutic relevance, presuming that similar in vivo observations are found [3,8].

## 4. Materials and Methods

### 4.1. Chemicals and Solution in This Work

QO-58 (5-(2,6-dichloro-5-fluoropyridin-3-yl)-3-phenyl-2-(trifluoromethyl)-1H-pyrazolol[1,5-a]pyrimidin-7-one, 5-(2,6-dichloro-5-fluoro-3-pyridinyl)-3-phenyl-2-(trifluoromethyl)-pyrazolo[1,5-a]pyrimidin-7(4*H*)-one, C_18_H_8_Cl_2_F_4_N_4_O) was supplied by Tocris (Union Biomed, Taipei, Taiwan), iberiotoxin and verruculogen (Ver) were by Alomone (Asia Bioscience, Taipei, Taiwan), while we acquired linopirdine, tetrodotoxin (TTX), and thyrotropin releasing hormone (TRH) from Sigma-Aldrich (Merck, Taipei, Taiwan). The stock solution of QO-58 was kept under −20 °C for long-term storage. Unless stated elsewhere, we obtained culture media (e.g., Ham’s F-12 medium), horse serum, fetal calf serum, L-glutamine, and trypsin/EDTA from HyClone^TM^ (Thermo Fisher, Tainan, Taiwan), and other chemicals such as CdCl_2_, aspartic acid, EGTA, and HEPES were of reagent grade.

The ion composition of extracellular solution (i.e., HEPES-buffered normal Tyrode’s solution) that we used in this work was as follows (in mM): NaCl 136.5, KCl 5.4, CaCl_2_ 1.8, MgCl_2_ 0.53, glucose 5.5, and HEPES-NaOH buffer 5 (pH 7.4). To record ionic flowing through macroscopic *I*_K(Ca)_, *I*_K(M)_ or *I*_K(erg)_, the patch electrodes were backfilled with the following intracellular solution (in mM): K-aspartate 130, KCl 20, MgCl_2_ 1, KH_2_PO_4_ 1, Na_2_ATP 3, Na_2_GTP 0.1, EGTA 0.1, and HEPES-KOH buffer 5 (pH 7.2). To measure *I*_K(M)_ or *I*_K(erg)_, we used a high-K^+^-bathing solution containing the following (in mM): KCl 145, MgCl_2_ 0.53, and HEPES-KOH 5 (pH 7.4). To record the activity of single K_M_ channels, the pipette solution was composed of the following (in mM): NaCl 136.5, KCl 5.4, MgCl_2_ 0.53, and HEPES-NaOH buffer 5 (pH 7.4), while to measure single BK_Ca_-channel activity, the pipette solution contained the following (in mM): KCl 145, MgCl_2_ 0.53, and HEPES-KOH 5 (pH 7.4). All solutions used in this work were prepared in deionized water from a Milli-Q^®^ water purification system (Merck Millipore, Taipei, Taiwan). For the purpose of being sterilized, we filtered the pipette solution and culture media with Acrodisc^®^ syringe filter which contains 0.2-μm Supor^®^ nylon membrane (#4612; Pall Corporation; Genechain, Kaohsiung, Taiwan).

### 4.2. Cell Preparations

The GH_3_ pituitary cell line, which was originally established from a pituitary tumor carried in a 7-month-old female Wistar-Furth rat, was supplied by the Bioresource Collection and Research Center (BCRC-60015; Hsinchu, Taiwan). This cell line was derived from the American Type Culture Collection (ATCC^®^ [CCL-82.1^TM^]; Manassas, VA, USA). We maintained GH_3_ cells in Ham’s F-12 medium supplemented with 2.5% heat-inactivated fetal calf serum (*v*/*v*), 15% horse serum (*v*/*v*) and 2 mM L-glutamine [62,64]. Cells were grown in monolayer culture at 37 °C in a humidified environment of CO_2_/air (1:19). For sub-culturing made by trypsinization (0.025% trypsin solution [HyClone^TM^] containing 0.01 sodium N,N-diethyldithiocarbamade and EDTA), we dissociated cells and then passaged them every 2–3 days. The measurements were undertaken when cell growth underwent 60–80% confluence (usually 5–6 days).

### 4.3. Electrophysiological Measurements

Shortly before the experiments, we carefully dissociated cells with a 1% trypsin/EDTA solution, and an aliquot of the suspension containing cell clumps was rapidly placed in a recording chamber adherently attached to the working stage of a DM-IL inverted microscope (Leica; Highrise Instrument, Taichung, Taiwan). The electrodes which were used to record were fabricated from Kimax-51^®^ capillaries with 1.5–1.8 mm in diameter (Kimble^®^ 34500-99; Merck, Taipei, Taiwan) by using a PC-10 vertical puller (Narishige; Taiwan Instrument, Tainan, Taiwan), and their tips were then fire-polished with MF-83 microforge (Narishige). When the electrodes were filled up with different internal solutions described above, their resistance was measured to range between 3 and 5 MΩ, for the purpose of making good GΩ-seal formation. We performed patch clamp recordings in cell-attached, inside-out or whole-cell configuration by using either an RK-400 (Bio-Logic, Claix, France) or an Axopatch-200B amplifier (Molecular Devices; Bestgen Biotech, New Taipei City, Taiwan), as elaborated elsewhere [29,31,52,59]. Whole-cell current recordings were established by rupturing the patch of membrane isolated with GW sealing by the patch pipette, then bringing the cell interior into contact with the pipette interior.

### 4.4. Data Recordings and Analyses

The signals were monitored and stored online in a Sony VAIO CS series laptop computer (VGN-CS110E; Tainan, Taiwan), equipped with a low-noise 1440A digitizer (Molecular Devices). During the measurements with analog-to-digital and digital-to-analog conversion, the latter device was controlled by pCLAMP 10.6 software (Molecular Devices) run under Microsoft Windows 7 (Redmond, WA, USA).

To assess the percentage increase of QO-58 on *I*_K(M)_ or *I*_K(Ca)_, we measured the amplitudes of *I*_K(M)_ or *I*_K(Ca)_ during cell exposure to different QO-58 concentrations (1–300 μM). The amplitude of *I*_K(M)_ or *I*_K(Ca)_ during cell exposure to QO-58 at a concentration of 300 μM was considered to be 100%, and the current amplitudes after application of different QO-58 concentrations were expressed relative to this value. The data sets with respect to concentration-dependent effect of QO-58 on the activation of *I*_K(M)_ or *I*_K(Ca)_ were satisfactorily fitted to the Hill equation with a nonlinear least-squares’ algorithm. Thus:

percentage increase (%)=(Emax×[C]nH)(EC50nH+[C]nH)

where [*C*] = the QO-58 concentration applied; *EC_50_* = the QO-58 concentration required for a 50% stimulation; *n_H_* = the Hill coefficient; and *E_max_* = the QO-58-induced maximal stimulation of *I*_K(M)_ or *I*_K(Ca)_.

The steady-state activation curve (i.e., the relationship of the membrane potential versus the *I*_K(M)_ conductance) acquired with or without exposure to QO-58 was satisfactorily approximated by a modified Boltzmann function of the following form:

GGmax=1{1+exp[−(V−V1/2)qF/RT]}

where *G* = the *I*_K(M)_ conductance; *G_max_* = the maximal conductance of *I*_K(M)_; *V_1/2_* = the voltage at which half-maximal activation of the current is achieved; *q* = the apparent gating charge; *F* = Faraday’s constant; *R* = the universal gas constant; and *T* = the absolute temperature.

The sigmoidal relationship between the membrane potentials and relative open-state probability of BK_Ca_ channels (i.e., the steady-state activation curve) with or without the QO-58 (3 μM) addition was collated and thereafter fitted by the Boltzmann equation using the goodness-of-fitness test;

(1) relative open probability=n{1+exp[−(V−V1/2)qF/RT]}

where *n* = the maximal relative open probability; *V* = the membrane potential; *V_1/2_* = the potential for half-maximal activation; *q* = apparent gating charge; and *F*, *R*, and *T* are similarly stated above in the activation curve of *I*_K(M)_.

### 4.5. Curve-Fitting Approximations and Statistical Analyses

Linear (e.g., single-channel conductance) or nonlinear (e.g., Hill or Boltzmann equation and single exponential) curves fitted to different experimental data sets were made with chi-squared goodness-of-fit test using either the Solver add-in bundled with Excel^®^ 2021 (Microsoft, Redmond, WA, USA)) or OriginPro^®^ 2021 (OriginLab, Scientific Formosa, Kaohsiung, Taiwan). The values are provided as means ± error of the mean (SEM) with the sizes of experimental observations, which represent the cell number sampled. The Student’s *t*-test (paired or unpaired) for two different group, or analyses of variance (ANOVA-1 or ANOVA-2) followed by post-hoc Fisher’s least-significance difference among more than two different groups studied for multiple comparisons, were made for the statistical evaluation. Probability with *p* < 0.05 was considered statistically significant (as indicated with * or ** in the figures), unless noted otherwise.

## Data Availability

The original data is available upon reasonable request to the corresponding author.

## Abbreviations

BK_Ca_ channel	large-conductance Ca^2+^-activated K^+^ channel
EC_50_	concentration required for 50% stimulation
*I-V*	current versus voltage
*I*_K(Ca)_	Ca^2+^-activated K^+^ current
*I*_K(erg)_	*erg*-mediated K^+^ current
*I*_K(M)_	M-type K^+^ current
K_M_ channel	M-type K^+^ channel
QO-58	5-(2,6-dichloro-5-fluoropyridin-3-yl)-3-phenyl-2-(trifluoromethyl)-1H-pyrazolol[1,5-a]pyrimidin-7-one/5-(2,6-dichloro-5-fluoro-3-pyridinyl)-3-phenyl-2-(trifluoromethyl)-pyrazolo[1,5-a]pyrimidin-7(4*H*)-one)
SEM	standard error of the mean
TRH	thyrotropin releasing hormone
TTX	tetrodotoxin
τ_act_	activation time constant
V_hys_	voltage-dependent hysteresis
V_ramp_	ramp voltage
